# Automated Imaging to Evaluate the Exogenous Gibberellin (Ga_3_) Impact on Seedlings from Salt-Stressed Lettuce Seeds

**DOI:** 10.3390/s24134228

**Published:** 2024-06-29

**Authors:** Mark Iradukunda, Marc W. van Iersel, Lynne Seymour, Guoyu Lu, Rhuanito Soranz Ferrarezi

**Affiliations:** 1 Department of Horticulture, University of Georgia, Athens, GA 30602, USA; mark.iradukunda@uga.edu (M.I.); mvanier@uga.edu (M.W.v.I.); 2 Department of Statistics, University of Georgia, Athens, GA 30602, USA; seymour@uga.edu; 3 College of Engineering, University of Georgia, Athens, GA 30602, USA; guoyu.lu@uga.edu

**Keywords:** *Lactuca sativa*, canopy size, crop modeling, germination, image analysis, salinity

## Abstract

Salinity stress is a common challenge in plant growth, impacting seed quality, germination, and general plant health. Sodium chloride (NaCl) ions disrupt membranes, causing ion leakage and reducing seed viability. Gibberellic acid (GA_3_) treatments have been found to promote germination and mitigate salinity stress on germination and plant growth. ‘Bauer’ and ‘Muir’ lettuce (*Lactuca sativa*) seeds were soaked in distilled water (control), 100 mM NaCl, 100 mM NaCl + 50 mg/L GA_3_, and 100 mM NaCl + 150 mg/L GA_3_ in Petri dishes and kept in a dark growth chamber at 25 °C for 24 h. After germination, seedlings were monitored using embedded cameras, capturing red, green, and blue (RGB) images from seeding to final harvest. Despite consistent germination rates, ‘Bauer’ seeds treated with NaCl showed reduced germination. Surprisingly, the ‘Muir’ cultivar’s final dry weight differed across treatments, with the NaCl and high GA_3_ concentration combination yielding the poorest results (*p* < 0.05). This study highlights the efficacy of GA_3_ applications in improving germination rates. However, at elevated concentrations, it induced excessive hypocotyl elongation and pale seedlings, posing challenges for two-dimensional imaging. Nonetheless, a sigmoidal regression model using projected canopy size accurately predicted dry weight across growth stages and cultivars, emphasizing its reliability despite treatment variations (*R*^2^ = 0.96, *RMSE* = 0.11, *p* < 0.001).

## 1. Introduction

Plant hormones are essential for regulating plant growth and development. They play a leading role in controlling and coordinating cell division, growth, and differentiation [1]. Though other factors and phytohormones play important roles in regulating germination and related processes, our study focused on the interaction of gibberellic acid (GA) and salinity stress. Among other plant hormones, GA and abscisic acid (ABA) are particularly important in regulating seed maturation, seed germination, and dormancy [2]. GA facilitates cell division and elongation, breaks dormancy, and speeds germination. On the other hand, ABA is key in inhibiting germination and establishing and maintaining seed dormancy, even under favorable conditions. ABA’s ability to interrupt the cell cycle allows it to inhibit germination [1]. The relationship between these hormones on seed germination is usually antagonistic, and their balance determines whether the seed will germinate [2,3,4,5]. In *Arabidopsis* seeds, when the GA/ABA ratio (more GA than ABA) is high during seed germination, there will be increased seed germination [3]. The opposite is also possible, where more ABA than GA in the seed during germination will reduce germination. During seed maturation, more ABA is desirable to prevent premature germination. This knowledge allows those involved in seed manipulation to inhibit or promote seed germination and/or seedling health.

Seed priming is widely used in crops to obtain uniform germination and high-quality seedlings. One such technique is the exogenous application of gibberellin (GA_3_) to seeds before or during sowing. This is possible because GA_3_ promotes seed germination and improves seedling vigor [6]. The application of GA_3_ to seeds of different crops significantly increased seed germination percentage and seedling vigor [7]. GA_3_ promotes seed germination and improves other aspects of plant performance, including plant height and shoot biomass [6]. However, higher GA_3_ concentrations can negatively impact germination rates [6]. It is also important to note that the optimum concentration and duration of GA_3_ treatment varies with different crops and cultivars. In addition, the effectiveness of plant hormones like GA can be affected by other factors. For example, studies conducted on wheat seeds found that GA metabolism-associated gene (GA_3_ox2) expression increases as the temperature rises [7]. In addition to promoting germination, GA_3_ can alleviate stresses on seeds and plants. This indicates an association between stress and an increase in GA metabolism. Another study [8] demonstrated that applying GA_3_ solution can promote seed germination even under high salinity stress. Salinity is one of the common plant stressors used in research studies. Salts like sodium chloride (NaCl) facilitate osmotic and ionic stress by altering membrane permeability, facilitating the excessive influx of sodium (Na^+^) and chloride (Cl_−_) ions. This ionic imbalance disrupts cellular homeostasis, increasing membrane depolarization and subsequent ion leakage. The resultant oxidative stress and metabolic perturbations contribute to reduced seed viability and impaired germination rates [9]. Salinity stress also hinders the water uptake needed by the seed to germinate properly [8]. At a hormonal level, salinity has been found to inhibit the synthesis of GA while promoting the synthesis of ABA. This will reduce the GA/ABA ratio and reduce germination [10]. 

GA_3_ has been observed to counteract the harmful effects of NaCl-induced salinity by promoting antioxidant activity and stabilizing cellular membranes. GA_3_ enhances seed germination and growth by mitigating oxidative stress and modulating the expression of stress-responsive genes. This hormonal intervention aids in the restoration of ion homeostasis and the maintenance of cellular integrity under salinity stress conditions [11,12]. A study with lettuce (*Lactuca sativa*) found that seeds under salt stress had highly reduced germination; however, by applying exogenous GA_3_, most seeds could recover and germinate [13]. When lettuce and arugula (*Eruca sativa*) plants received GA_3_, they increased their tolerance to salinity stress [14]. It is clear that applying GA_3_ to seeds improves their germination and health. But the question remains: do those early variations due to GA_3_ and salt stress in germination and plant growth from seed handling persist until the end of the plant’s life cycle? To answer that question, it is necessary to follow the growth habit of plants from seed to harvest. When collecting continuous data on plant growth, recent developments in non-invasive imaging techniques are very promising. As described in multiple studies, low-cost camera systems can quantify the growth of lettuce and detect plant health-related issues [15,16,17,18,19]. While more work is needed to improve the adaptability of vision-based tools, we tried to integrate cameras in controlled environment production systems. The system can provide real-time data on plant performance in response to induced variations due to salinity stress and exogenous GA_3_.

Our study aimed to use an automated imaging system and crop modeling to measure the impact of soaking lettuce seeds in a solution containing NaCl and GA_3_ on seed germination and seedling canopy size. The hypothesis speculated that lettuce seeds subjected to salt stress (NaCl) would experience reduced germination. Furthermore, introducing an exogenous GA_3_ solution to salt-stressed seeds was anticipated to enhance germination and seedling canopy size, with superior outcomes expected at higher concentrations of GA_3_ as detected by our imaging system.

## 2. Materials and Methods

### 2.1. Location and Environmental Conditions

The research took place at the University of Georgia (College of Agricultural and Environmental Sciences, Department of Horticulture, Controlled Environment Agriculture lab) in Athens, GA, USA (latitude 33°57′26.676″ N, longitude 83°22′36.48″ W). Plants were cultivated in a large walk-in growth chamber. The environmental conditions of the experiment were as follows: temperature 25 °C, relative humidity 70%, vapor pressure deficit 0.91 kPa, carbon dioxide 800 mg/L, and light intensity 250 µmol·m^2^·s^−1^. The chamber provided a 16 h light cycle or photoperiod from 12 a.m. to 4 p.m., equating to a daily light integral of 14.4 mol·m^−2^·d^−1^. Daily watering was carried out with a 10N-2.2P-12.4K nutrient solution containing 100 mg/L nitrogen (15-5-15 Ca-Mg Professional LX; J.R. Peters, Allentown, PA, USA) through an automated ebb-and-flow subirrigation system that ran for 5 min daily.

### 2.2. Seed Treatments

**Figure 1:**
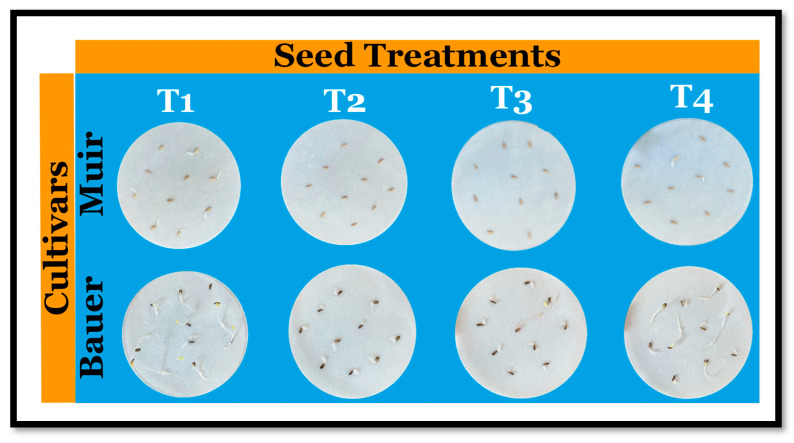
Visual representation of seed treatment arrangements for lettuce (*Lactica sativa*) cultivars on filter papers, where T1: distilled water (control), T2: 100 mM sodium chloride (NaCl), T3: 100 mM NaCl + 50 mg/L gibberellin (GA_3_), and T4: 100 mM NaCl + 150 mg/L GA_3_.

We selected two green-colored lettuce cultivars, ‘Bauer’ and ‘Muir’ (Jonny’s Selected Seeds, Winslow, ME, USA). Most of the experimental procedure was adopted from [13]. For solutions, we used sodium chloride (NaCl granular; Thermo Fisher Scientific, Waltham, MA, USA) and GA_3_ powder with 90% purity (Gibberellic Acid 90%; Power Grown, Rocklin, CA, USA). The salt solution was prepared by dissolving NaCl in distilled water to make 100 mM (~5844 mg/L). We dissolved GA_3_ powder first in 2 mL of 70% ethanol (GA_3_ powder dissolved better in alcohol) and then added distilled water to make 1000 mL. Care was taken to prevent GA_3_ exposure to sunlight. Four treatment (T) solutions were made: T1 (distilled water or dH_2_O), T2 (100 mM NaCl), T3 (100 mM NaCl + 50 mg/L GA_3_), and T4 (100 mM NaCl + 150 mg/L GA_3_) (Figure 1). Filter papers were wetted with 2 mL of each solution, and the extra solution was removed. 

We carefully placed seeds on the wetted filter papers and covered the Petri dish. Each treatment was repeated twice with 15 seeds per Petri dish. The Petri dishes were placed at once in a growth chamber (CMP3244; Conviron-Controlled Environments Limited, Winnipeg, MB, Canada) and kept in the dark for 24 h at 25 °C.

### 2.3. Experimental Setup, Image Acquisition, and Image Analysis

After 24 h, the seeds that showed a protruded radicle were sown and grown in a walk-in chamber. We used embedded computers with cameras to collect colored (RGB) images daily from sowing to maturity. Images were analyzed to collect projected canopy size (PCS) and germination rate. The growing conditions, image analysis, and data analysis methods we used were the same as in our previous study [15].

## 3. Results

### 3.1. Projected Canopy Size over Time

**Figure 2:**
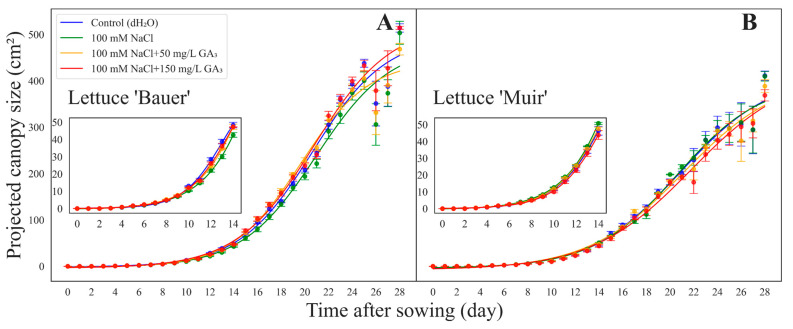
The curves showing a relationship between the projected canopy size (PCS) of lettuce (*Lactuca sativa*) seedlings of each seed treatment of cultivar (**A**) ‘Bauer’ and (**B**) ‘Muir’ and time (days after sowing) obtained by embedded computers. PCS data were collected every day from sowing to 14 days. Each data point and the error bars indicate the mean and standard deviation of 16 seedlings at each day in each treatment/cultivar. Inserts display PCS for the seedling stage (initial 14 days after sowing).

In both lettuce cultivars, the PCS increased over time in all treatments (Figure 2). No germination was observed until day four, followed by a gradual increase in PCS from day five to seven. Subsequently, both cultivars showed an exponential growth phase, followed by a plateau phase, forming a sigmoidal (S-shaped) growth curve (Figure 2A,B). Although no significant differences were observed amongst treatments in the seedling stage, the pattern remained the same throughout the growth cycle: the best-performing plants continued to be so until the final harvest (28 days after sowing).

**Figure 3:**
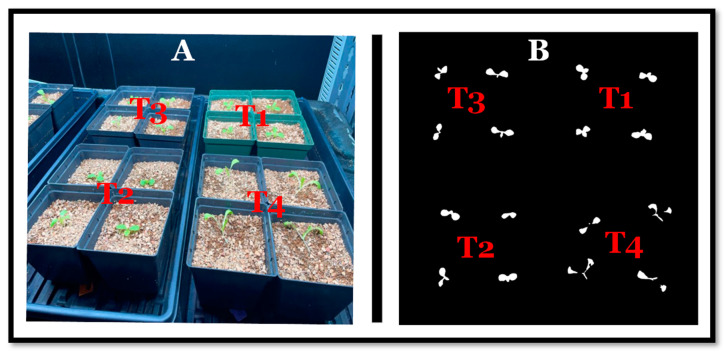
Images showing different views of the seedlings. (**A**): Side view image taken by a cell phone. (**B**): Camera view of the analyzed images on the same day, where T1: distilled water (control), T2: 100 mM sodium chloride (NaCl), T3: 100 mM NaCl + 50 mg/L gibberellin (GA_3_), and T4: 100 mM NaCl + 150 mg/L GA_3_.

Seven days after sowing, we observed that the seedlings from seeds treated with high GA_3_ (T4) had extended leaves and hypocotyls and looked taller than other plants (Figure 3). Since our cameras took top-view (two-dimensional) images, the results did not reflect what was happening. First, when the leaves were floppy, they were likely not as exposed to the camera. Secondly, the hypocotyls were whiter and were not counted as plant material by the threshold we used in our imaging technique. As a result, the leaves in T4 appeared detached from the plant, causing an underestimation of the canopy size (Figure 3).

### 3.2. Germination Curves

**Figure 4:**
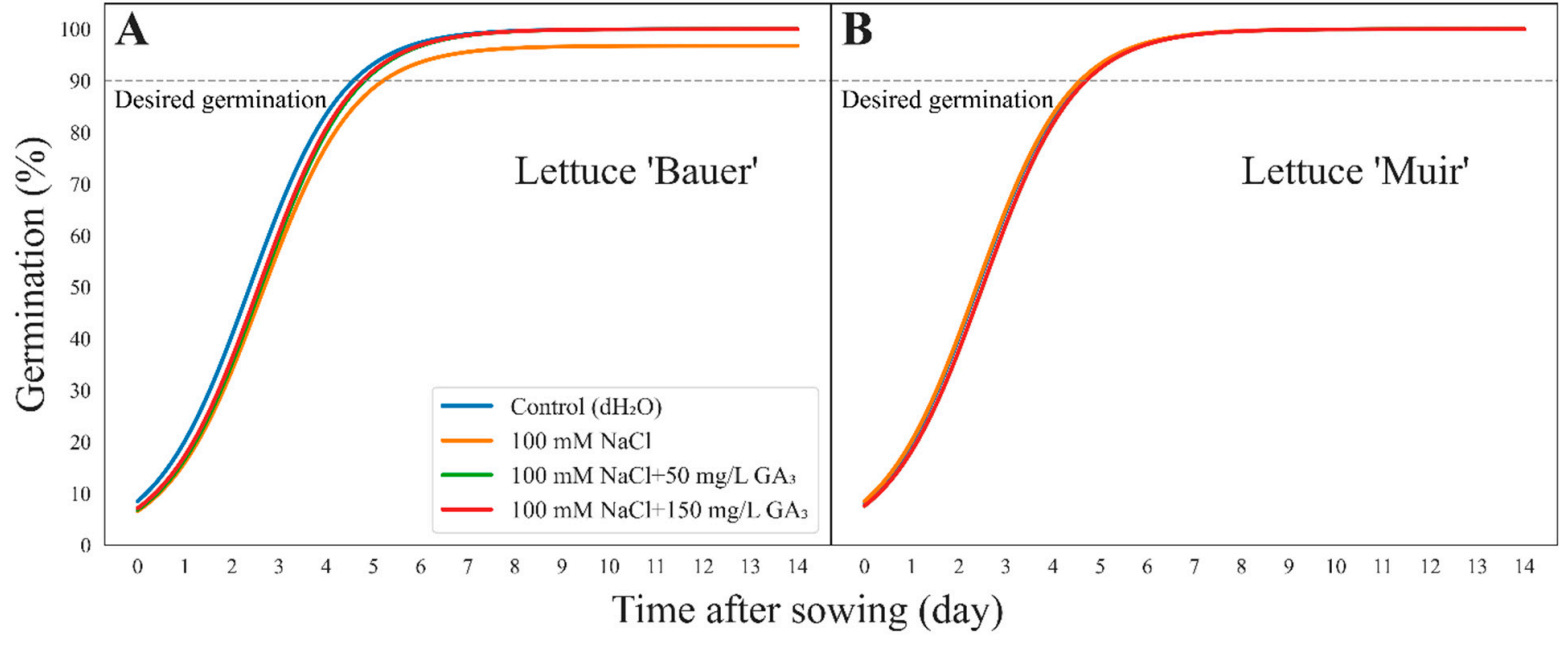
Sigmoidal curves showing germination percent over time colored by seed treatments in lettuce (*Lactuca sativa*) cultivar (**A**) ‘Bauer’ and (**B**) ‘Muir’. The germination percentage was calculated from data obtained by embedded computers. The grey dashed line shows the desired germination (90%) and where the curve did not reach the line means poor germination.

Germination percentages exhibited minimal variations among lettuce cultivars and treatments (Figure 4). Seeds soaked in 100 mM NaCl displayed slower germination, while the control (dH_2_O) showed the fastest, aligning with expectations (Figure 4A). It took the control (dH_2_O), 100 mM NaCl + 50 mg/L GA_3_, and 100 mM NaCl + 150 mg/L GA_3_, about four days to reach 90% germination, while it took five days for the seeds soaked in 100 mM NaCl. The cultivar ‘Muir’ displayed consistent germination behavior across treatments, reaching 90% germination by day five (Figure 4B). 

### 3.3. Shoot Dry Weight

**Figure 5:**
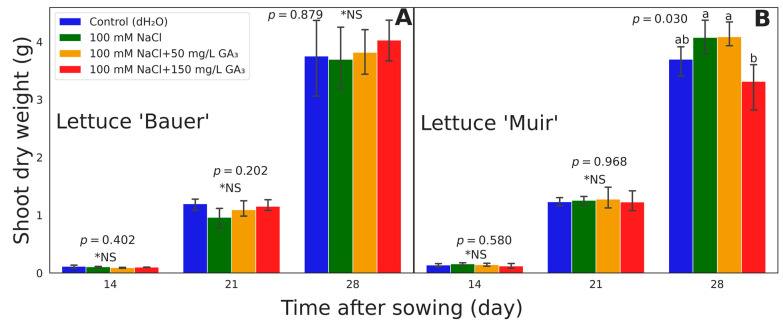
Shoot dry weight collected at 14, 21, and 28 days after sowing of lettuce (*Lactuca sativa*) cultivars (**A**) ‘Bauer’ and (**B**) ‘Muir’. Each bar and the error bars indicate the mean and standard deviation of 32 seedlings in each treatment. * NS in each harvest denotes a non-significant difference, and values followed by the same letter are not significantly different according to Tukey’s HSD at α = 0.05.

For the cultivar ‘Bauer’, no significant treatment variations were observed in shoot dry weight collected on day 14 after sowing (*p* = 0.402), day 21 (*p* = 0.202) and final shoot dry weight on day 28 (*p* = 0.879) (Figure 5A). The average weight for the control (dH_2_O), 100 mM NaCl, 100 mM NaCl + 50 mg/L GA_3_, and 100 mM NaCl + 150 mg/L GA_3_, was 0.113, 0.106, 0.090, and 0.100 g on day 14; 1.197, 0.962, 1.092, and 1.152 g on day 21; and 3.753, 3.695, 3.818, and 4.028 g on day 28, respectively (Figure 5A).

There were no significant differences between treatments on the cultivar ‘Muir’ (*p* = 0.580) in shoot dry weight collected on days 14 and 21 after sowing (*p* = 0.968). Conversely, significant differences were noted for the cultivar ‘Muir’ on day 28 (*p* = 0.030) (Figure 5B). The seeds soaked in 100 mM NaCl + 50 mg/L GA_3_ with 3.305 g at 28 days after sowing exhibited the highest shoot dry weight. The average weight for the control (dH_2_O), 100 mM NaCl, 100 mM NaCl + 50 mg/L GA_3_, and 100 mM NaCl + 150 mg/L GA_3_, was 0.110, 0.126, 0.115, and 0.096 g on day 14; 0.995, 1.015, 1.032, and 0.992 g on day 21; and 2.990, 3.295, 3.305, and 2.680 g on day 28, respectively (Figure 5B). Multiple comparisons revealed significant differences between treatments on day 28 after sowing (Tukey’s HSD, α = 0.05), where values that share similar letters are not significantly different (Figure 5).

### 3.4. PCS and Shoot Dry Weight

**Figure 6:**
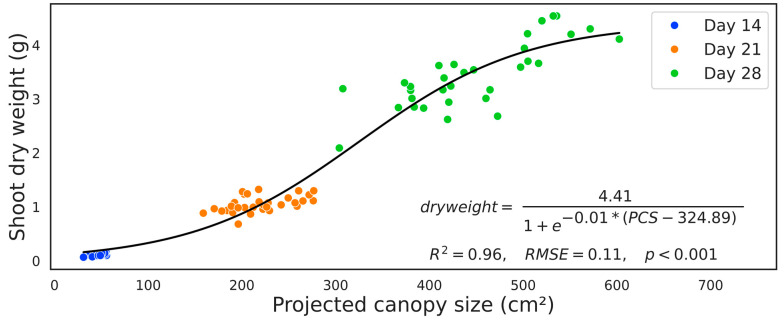
The relationship between projected canopy size and corresponding shoot dry weight colored by days of harvest (to collect shoot dry weight). The black solid line shows a sigmoidal regression with an equation, regression summaries of coefficient of determination (R^2^), root mean square error (RMSE), and *p*-value (*p*).

Combining all canopy size data with all three harvests revealed that canopy size was a better predictor of dry weight (*t* = 39.099, *p* < 0.001). The cultivar had no significant effect on shoot dry weight (*t* = 1.555, *p* = 0.123), and seed treatments had no significant impact (*t* = 1.045, *p* = 0.299). A sigmoidal regression model (generalized for all three harvests) showed a strong positive relationship between PCS and the shoot dry weight (*R*^2^ = 0.96, *RMSE* = 0.11, *p* < 0.001). However, the model overestimates values for young plants at day 14 as all the data points are below the regression line (Figure 6). The regression equation shows a growth rate of 0.01 g/cm^2^ with a maximum dry weight of 4.41 g. The half-maximum growth rate was reached when PCS was 324.89 cm^2^.

## 4. Discussion

The general trend for all treatments and cultivars suggested a positive development in plant canopy size throughout the experiment without any significant differences (Figure 2A,B). Seeds soaked in 100 mM NaCl demonstrated the slowest germination, while the control (dH_2_O) exhibited the fastest germination, aligning with our expectations (Figure 4A). Seeds treated with 100 mM NaCl showed diminished germination because NaCl ions negatively affect the membrane activity chemically (osmotic pressure) and physically (water uptake), which hamper the germination process in its early stages [8]. Salinity, primarily due to NaCl, threatens seed germination, seedling vigor, and overall plant health by inducing osmotic stress, ion toxicity, and oxidative damage. High concentrations of NaCl reduce the osmotic potential of the soil, making it difficult for seeds to absorb water, which is crucial for germination. This leads to a significant decline in germination rates and delays in germination time, severely affecting early seedling establishment and vigor [9,14]. Salinity disrupts ion homeostasis within plant cells by causing an accumulation of toxic ions like Na^+^ and Cl^−^, which can interfere with essential metabolic processes, leading to nutrient imbalances and reduced photosynthetic efficiency. This ionic imbalance impairs root and shoot growth, resulting in stunted seedlings with poor vigor [12]. Additionally, the excess salt triggers the production of reactive oxygen species or ROS, causing oxidative stress that damages cellular components such as lipids, proteins, and nucleic acids, further compromising plant health and productivity [9,12]. This is why we observed a decrease in germination when seeds were stressed with salt. The germination of the cultivar ‘Bauer’ seeds stressed with salt was improved by GA_3_ application (Figure 4A). A suggested hypothesis is that the GA_3_ helps the seed absorb water for germination by repairing the membrane damaged by salt stress [8]. These results are comparable to other studies where seed germination and seedling growth were significantly improved by applying exogenous GA_3_ on the salt-stressed seeds [8,13]. Another negative impact of salinity on seeds is that there will be a toxic accumulation of hydrogen peroxide or H_2_O_2_. For germination to occur, the seed must be able to degrade poisonous elements using scavenging enzymatic activity. The increased gene expression of enzymes responsible for detoxification (*HvBAS1* and *HvMT2*) due to the presence of GA explains how exogenous GA_3_ helped barley seeds recover from salinity and other stressors [8]. For example, temperature-damaged ‘Hwahong’ lettuce seeds could recover their germination by receiving 100 mg/L GA_3_ [20]. 

GA_3_ ameliorates the detrimental effects of salt stress on lettuce seeds through several critical pathways. Under saline conditions, GA_3_ enhances the selectivity and efficiency of ion transport, thereby reducing the toxic buildup of Na^+^ and Cl^−^ ions within cells, which helps to maintain osmotic balance and prevent ionic toxicity. Moreover, GA_3_ upregulates the activity of key antioxidant enzymes like superoxide dismutase and catalase, which neutralize reactive oxygen species or ROS generated by salt stress, thus mitigating oxidative damage to cellular components [14,21,22]. Additionally, GA_3_ plays a significant role in stabilizing cell membrane integrity by influencing the composition and synthesis of membrane lipids and proteins, which decreases ion leakage and maintains cellular homeostasis [23]. GA_3_ also modulates the expression of stress-responsive genes, thereby enhancing the plant’s ability to cope with salinity-induced stress at a genetic level [24]. Furthermore, GA_3_ supports seed germination and seedling growth by promoting cell elongation and division, counteracting the inhibitory effects of high salinity [25]. Finally, GA_3_’s interaction with other plant hormones, such as ABA, balances growth and stress responses, thereby optimizing the overall adaptive capacity of the plant under salt stress [21].

Beyond mitigating stress, GA supports optimal plant performance to increase yield. GA_3_ improved the stomatal conductance and accumulation of sugars in lettuce and arugula, which increased dry mass [22]. However, the authors caution that GA_3_ effects, like other phytohormones, will differ in plant species, concentrations, and application timing [22]. We saw similar results where 100 mM NaCl + 150 mg/L GA_3_ resulted in the lowest final shoot dry weight in the cultivar ‘Muir’ (Figure 5B). We theorize that high levels of GA_3_ (>100 mg/L) can damage this cultivar ‘Muir’ or the seeds were damaged beyond recovery. However, we cannot verify this because we did not perform any seed molecular analysis to see what changes could have yielded this response in this cultivar. In another study, when high levels of GA_3_ were applied to lettuce via a solution in a hydroponic system, negative effects, including pale leaves, were shown [22]. Seven days after germination in all cultivars, GA_3_-treated seedlings showed extended hypocotyls and floppy leaves, while the control had more expanded leaves and was closer to the substrate (Figure 3). The expanded hypocotyl in the presence of exogenous GA_3_ can be attributed to GA_3′_s ability to promote cell expansion [1,8]. Another study showed that GA_3_ reversed the negative impact of salinity stress in seeds by promoting phosphate transport and increasing the shoot length [13]. Though plants from GA-treated seeds showed extended leaves and hypocotyl, the shoot dry weight analysis revealed no significant treatment variations for the cultivar ‘Bauer’ on all days (Figure 5A). In the same way, there were significant differences only on day 28 for the cultivar ‘Muir’ (Figure 5B). Due to the height between shelves (reduced distance between camera and plants) and camera view limitations, we could only grow plants for up to 30 days. Since the cultivar ‘Muir’ is slow growing, allowing it to reach maturity (~50 days from sowing) might have provided more weight variations.

Despite those physical limitations, the sigmoidal regression model demonstrated a sigmoidal relationship between PCS and shoot dry weight across all harvests (*R*^2^ = 0.96, *RMSE* = 0.11, *p* < 0.001), emphasizing the predictive nature of canopy size on plant growth (Figure 6). Similar studies demonstrated the significance of canopy size in predicting dry weight [26,27,28]. Inaccuracies in projecting canopy size can be attributed to variations in leaf thickness over time as plants mature [29]. In addition to leaf thickness, the top-view imaging can only capture top leaves, so we could not capture the whole plant area. This caused our model to overestimate smaller plants and underestimate the dry weight of bigger plants. As a solution, more descriptive images can be obtained by taking pictures from various angles of the plant [27].

## 5. Conclusions

The experiment demonstrated the overall negative effect of salinity stress on seed germination and early seedling vigor. While the cultivar ‘Bauer’ showed no significant treatment differences in shoot dry weight, the cultivar ‘Muir’ exhibited variations, especially in the final harvest, where GA_3_-treated seeds did poorly, contrary to our hypothesis. The variations of GA_3_ effects on lettuce cultivars reinforce how plant hormone activity depends on species, dose (concentration and exposure time), and the plant growth stage. Nonetheless, applying GA_3_ to stressed seeds or young seedlings improved germination and plant size. It emphasizes the potential of exogenous GA_3_ in improving other plant behaviors. The strong correlation between canopy size and shoot dry weight underscores the importance of monitoring canopy development for predicting plant growth regardless of salinity stress and GA_3_ treatments and lettuce cultivars. Using automated and cheaper imaging tools installed in a growth chamber, we could pick out differences in plant growth under those variations. However, care in using two-dimensional canopy data to predict dry weight should be taken when the plant morphology features are out of the ordinary, like floppy leaves or plants growing more vertically than expected. The easier solution so far has been to take images from multiple angles. However, this is more appropriate for analyzing a single plant and would add to the complexity of camera installation in a vertical farm where other activities occur. We suggest that, where possible, increasing the distance between the camera and the plants and growing more uniform plants would help.

## Data Availability

Dataset available on request from the authors.
